# Vine Shoots as a Source of *Trans*-Resveratrol and *ε*-Viniferin: A Study of 23 Italian Varieties

**DOI:** 10.3390/foods11040553

**Published:** 2022-02-15

**Authors:** Mirella Noviello, Antonio Francesco Caputi, Giacomo Squeo, Vito Michele Paradiso, Giuseppe Gambacorta, Francesco Caponio

**Affiliations:** 1 Department of Soil, Plant and Food Science (DISSPA), University of Bari Aldo Moro, Via Amendola, 165/a, I-70126 Bari, Italy; mirella.noviello@uniba.it (M.N.); antonio.caputi1@uniba.it (A.F.C.); giacomo.squeo@uniba.it (G.S.); giuseppe.gambacorta@uniba.it (G.G.); 2 Department of Biological and Environmental Sciences and Technologies, University of Salento, S.P. 6, Lecce-Monteroni, I-73100 Lecce, Italy; vito.paradiso@unisalento.it

**Keywords:** stilbene, vine shoots, viticulture waste, *trans*-resveratrol, ε-viniferin, Italian varieties

## Abstract

Stilbenes are a family of phenolic secondary metabolites that are known for their important roles in plant protection and human health. Numerous studies show that vine shoots, one of the most abundant winery wastes, could be used as a source of bioactive compounds such as stilbenes. The predominant stilbenoids in vine shoots are *trans*-resveratrol (Rsv) and *ε*-viniferin (Vf), whose content varies depending on numerous intrinsic and extrinsic factors. The present work investigates the influence of pre-treatment and variety on stilbene concentration in vine shoots. Vine shoots of the Primitivo and Negroamaro varieties were submitted to four different trials before stilbene extraction (untreated, dried at 50 °C for 24 h, dried at 70 °C for 15 min, and dried at 80 °C for 10 min). The results showed that the heat pre-treatments had a slight impact on the total phenol and stilbene content. In contrast, the variety variable had a stronger impact on stilbene concentration, ranging from 2700 to 6400 mg kg^−1^ DW for untreated vine shoots of 23 Italian varieties. In all vine shoots, the most abundant stilbene compound was Rsv and the highest content was found in vine shoots of the Nero di Troia (5298.1 mg kg^−1^ DW) and Negroamaro (5249.4 mg kg^−1^ DW) varieties.

## 1. Introduction

Stilbenoids are a natural family of polyphenolic compounds that exist both as monomers and as oligomers, with a diphenyl ethylene group oriented in cis or trans configurations [1]. These compounds have gained interest not only for their several biological activities, but also for their complex structural conformation [2]. Numerous studies show that the beneficial properties of stilbenes for human health include protective effects against cancer (as they inhibit cell proliferation) [3], diabetes [4], neurodegenerative diseases such as Alzheimer’s disease [5], and coronary heart disease [6]. They are also used as multifunctional ingredients in cosmetics [7]. Recently, the possibility of developing drugs against COVID-19 using natural stilbene compounds has been evaluated [8]. In addition, stilbenes are used in agriculture as alternative anti-phytopathogenic substances [9,10].

Stilbenes are mainly synthesized by plants as phytoalexins in response to biotic and abiotic stress (e.g., pathogens, ultraviolet irradiation, heavy metal ions, mechanical damage, frost, thermal treatment, or ozone) [11]. Their distribution is very heterogeneous in the plant kingdom [12]. In fact, stilbenes have been isolated and identified in at least 72 plant species belonging to 31 genera and 12 families, including *Vitaceae,* in which these compounds are present in lignified stem tissue, in grape berries and in wines [11,13,14]. Several reviews have indicated that winery wastes and by-products are rich in stilbenes, which have been extracted and applied in multiple fields based on their beneficial properties [15,16].

The cultivation of vines is widespread: in 2020, the world area under vine cultivation for all purposes (wine and juices, table grapes, and raisins) was estimated at 7.3 million hectares (Mha), of which 3.3 Mha are in the European Union. Italy has an area under vine cultivation of 719 thousand hectares, an increase of over 0.8% from 2019 [17]. Consequently, the wine-growing sector produces many and various wastes, generated from agricultural practices (e.g., vine shoots, leaves, stems) as well as from the winemaking process (e.g., grape stalks, pomace, wine lees). In particular, vine shoots (also called grapevine canes) are the most significant vine waste material from a quantitative point of view, with a weight of 2–5 tonnes per hectare per year, depending on density of plantation, climate, vigour of the vine, and other agronomical factors [18].

Vine shoots have a very low economic value; in fact, they are burned [19] or incorporated into the soil to promote the degradation of organic matter and reduce the need for organic fertilizers [20]. Some other possible applications of this material include the production of pulp paper [21], solid biofuels [22], or the possibility of obtaining activated carbon [23]. Recently, attention has shifted to the possibility of using vine shoots in the agri-food industry, in a circular economy perspective. One of the possible applications studied is their use as an alternative to oak chips as an enological coadjuvant to improve the sensorial profile of wines, [24,25]. Moreover, recent studies have shown that vine shoots are rich in bioactive compounds, such as stilbenes, that make this by-product an untapped source of these compounds with important antioxidant, anti-microbial, and anti-aging properties and multiple possible applications [15]. Up to 41 stilbenes have been found in vine shoots and among these, *trans*-resveratrol (Rsv) and *ε*-viniferin (Vf) are the most abundant [15,26]. Several studies tested stilbene-enriched vine shoot extracts as a preservative in wine in order to reduce the use of SO_2_ in winemaking [15,27].

The concentration and composition of stilbenes in vine shoots are subject to extreme variability due to many intrinsic and extrinsic factors. These factors include the variety and geographical area of origin [28,29,30], vineyard age [31], or climate conditions [32]. Considering the variety analysed in literature, vine shoots of Pinot Noir and Gewurztraminer have been reported as those with the highest content of stilbenes [15,18,29,30]. On the other hand, the extrinsic factors include the extraction method [26], storage time and temperature of the vine shoots, or various pre-treatments, such as the cutting length or thermal treatments, before stilbenes extraction [32,33,34,35,36,37,38]. Despite the available reports, the literature does not clarify univocally the effects of these heat treatments on stilbene quantities [26,39,40]. Moreover, it is well known that the low-temperature/long-time heat treatments, mostly adopted for vine shoots, generally led to a higher reduction of the nutritional values of foods than the high-temperature/short-time heat treatments [41]. A previous work proved that treatments applied to Coratina olive cultivar leaves at high temperatures and short times did not cause a reduction of the phenolic compounds [42]. Consequently, investigations on the effect of the temperature-time conditions are necessary to preserve these compounds and increase the extraction yields.

The aim of this study was twofold: (i) select the most appropriate vine shoots treatment before stilbene extraction (untreated, dried at 50 °C for 24 h, dried at 70 °C for 15 min, dried at 80 °C for 10 min) using two testing varieties (Primitivo and Negroamaro); (ii) study the variability of the total phenolic content and the Rsv and Vf amounts of vine shoots from 23 Italian varieties. To the best of our knowledge, the stilbene contents of vine shoots from these Italian varieties has not been studied yet.

## 2. Materials and Methods

### 2.1. Plant Materials

Vine shoots of 23 varieties of *Vitis vinifera* L. were selected: Aglianico (AG), Bianco d’Alessano (BA), Bombino Bianco (BB), Bombino Nero (BN), Ciliegiolo (CI), Fiano Bianco d’Avellino (FB), Italia (IT), Malvasia Bianca (MB), Malvasia Nera di Brindisi (MN), Maresco Bianco (MA), Minutolo Bianco (MI), Montepulciano (MO), Negroamaro (NE), Nero di Troia (NT), Notardomenico (ND), Ottavianello (OT), Palieri (PA), Primitivo (PR), Sangiovese (SA), Susumaniello (SU), Trebbiano (TR), Verdeca (VE), and Vittoria (VI). All vine shoots were sampled during winter (February 2021) from a varietal collection located in Locorotondo (Puglia, Italy; coordinates: longitude 17°13′3.741″ E, latitude 40°45′42.763″ N) grown under the same conditions. The vineyard was planted in 1985 on a sub-alkaline medium-textured soil. About 10 kg of vine shoots from each variety, sampled from large batches and representative of these, were collected and stored intact under controlled conditions (darkness, at 15 ± 3 °C) for 6 weeks [34]. Then, two different representative subsamples of about 1 kg for each variety were considered for the subsequent analyses. Table 1 shows additional information about the varieties chosen in this work.

#### 2.1.1. Evaluation of Treatments Impact

Vine shoots from the Primitivo and Negroamaro varieties were manually cut (particle size about 5 cm long), cut crosswise, heat-treated (as described below), ground (particle size ranging from 0.2–4 mm) using a hammer mill (Dietz-Motoren KG, Elektromotorenfabrik, 7319 Dettingen-teck, Germany), and immediately submitted to extraction and analyses. Four different treatments of the vine shoots were tested before stilbene extraction (untreated, dried at 50 °C for 24 h, dried at 70 °C for 15 min, dried at 80 °C for 10 min). A thermostatic oven (TFC 120 forced air oven, ArgoLab) was used for the drying process. The moisture content of the vine shoots was measured using a thermobalance (Ladwag MAC 110/NP, Radwag, Poland).

#### 2.1.2. Evaluation of Variety Impact

The stilbene contents of the untreated vine shoots from 23 Italian varieties were assessed. The vine shoots were manually cut (particle size around 5 cm long), cut crosswise, ground (particle size ranging from 0.2–4 mm) using a hammer mill (Dietz-Motoren KG, Elektromotorenfabrik, 7319 Dettingen-teck, Germany), and immediately submitted to extraction and analyses. The moisture content of the vine shoots was measured using a thermobalance (Ladwag Mac 110/NP, Radwag, Poland).

### 2.2. Extraction Procedure

The extraction of the stilbenes from the vine shoots was carried out according to Vergara et al. [29], with some modifications. Briefly, an aliquot of vine shoots (2 g) was added with 16 mL of an ethanol/water solution (80:20 *v*/*v*) and sonicated in an ultrasonic bath (CP104 Standard Ultrasonic Cleaning Machine, CEIA, Padova, Italy) at room temperature and 50 Hz for 5 min. The extract was centrifuged (SL 16R Centrifuge, Thermo Scientific, MA, USA) at 10,000× *g* for 5 min, the supernatant was separated, filtered through Whatman filter paper (GE Healthcare, Milan, Italy) (67 g m^−2^), and then filtered using nylon filters of 0.45 µm (Sartorius Stedim Biotech Gmbh, Göttingen, Germany) and used for chemical characterization. Extractions were carried out in duplicate for each condition tested.

### 2.3. Extract Characterization

#### 2.3.1. Total Phenolic Content Determination

The total phenol content was determined according to the Folin–Ciocalteu method [43]. To 980 μL of H_2_O Milli-Q, 20 μL of appropriately diluted extract, 100 μL of Folin–Ciocalteu reagent were added. After 3 min, 800 μL of 7.5% Na_2_CO_3_ were added and then the sample was stored in the dark for 60 min. The absorbance was read at 720 nm (Cary 60 UV-Vis, Agilent Technologies, Mulgrave, Australia). The results were expressed as mg of gallic acid equivalents (GAE) per g of dry weight sample (mg GAE g^−1^ DW). Each sample was analysed in duplicate.

#### 2.3.2. Antioxidant Activity Evaluation

The DPPH (2,2-diphenyl-1-picrylhydrazyl) assay was performed on the extracts according to the procedure of Tarantino et al. [44]. Each extract (50 µL) was combined with 950 µL DPPH solution (0.08 mM in ethanol). The decrease in absorbance was read at 517 nm using a Cary 60 UV-Vis spectrophotometer (Agilent, Cernusco, Milan, Italy). The results were expressed in µmol Trolox equivalents g^−1^ dry weight for all vine shoot samples (µmol TE g^−1^ DW). All determinations were carried out in duplicate. Antioxidant activity was also determined by ABTS-TEAC assay [44]. For spectrophotometry, the reaction took place directly in cuvettes by adding 50 µL of each sample to 950 µL of final ABTS^•+^ solution. After 8 min, the decrease in absorbance was measured at 734 nm, using a Cary 60 UV-Vis spectrophotometer (Agilent, Cernusco, Milan, Italy). The results were expressed in µmol TE g^−1^ dry weight for all vine shoot samples (µmol TE g^−1^ DW). Each sample was analysed in duplicate.

#### 2.3.3. Quantification of Rsv and Vf by HPLC-DAD

The analysis of the stilbenes was performed according to the method of Ewald et al. [38] using high-performance liquid chromatography (UltiMate 3000 HPLC, Thermo scientific, Munich, Germany) that included an HPG-3200RS binary pump, WPS-3000RS/TRS autosampler, TCC-3000RS column oven, and a DAD-3000RS photodiode array detector. HPLC separation was achieved on Acclaim^TM^ 120 C18 columns (120 Ǻ 3 × 150 mm, 3 μm) maintained at 25 °C using a mobile phase consisting of 1% aqueous acetic acid (*v*/*v*) (A) and methanol (B). The separation was carried out at 25 °C with a flow rate of 0.6 mL min^−1^ under the following conditions: 0 min (20% B), 10 min (20% B) 6.5 min (37% B), 12.6 min (50% B), and 21.0 min (100% B). Under these conditions, Rsv and Vf were eluted with a retention time of 14.7 min and 17.8 min and monitored at 306 and 324 nm, respectively. Calibration curves were prepared using the endotoxin standards (Sigma-Aldrich, Steinheim, Germany) of Rsv (R^2^ = 0.9993) and Vf (R^2^ = 0.9994) in the concentration range 1–500 mg L^−1^. The amount of Rsv and Vf found in each extract was expressed as mg of compound kg^−1^ of DW. Each sample was analysed in duplicate.

### 2.4. Statistical Analysis

Minitab17 (Minitab Inc., State College, PA, USA) was used for the statistical analysis of all results, reported as mean ± standard deviation (SD) of two replications. To evaluate the differences between samples, one-way ANOVA was applied. The Fisher LSD test was employed for the post-hoc comparisons of the means. Correlation between variables was determined by Pearson’s correlation coefficient test. Statistical significance was set at *p* < 0.05 level.

## 3. Results and Discussion

### 3.1. Evaluation of the Pre-Treatments

#### 3.1.1. Total Phenolic and Antioxidant Activity

Table 2 reports the mean values, standard deviation, and results of the statistical analysis of the total phenolic contents and the antioxidant activity measured in the vine shoot extracts subjected to the different investigated treatments. Several studies have shown that vine shoots are rich in phenolic compounds [45,46,47,48,49]. Concerning the vine shoots of the Primitivo variety, the results showed that the extracts, irrespective of the pre-treatments, contained similar amounts of TPC, except for vine shoots treated at 50 °C for 24 h, in which a significant reduction was observed (18.4 ± 0.1 mg g^−1^ DW). Instead, in regards the vine shoot extracts of the Negroamaro variety, except for the treatment at 80 °C for 10 min, the other two applied heat treatments reduced the TPC. In particular, the treatment at 70 °C for 15 min reduced TPC by 11.3% with respect to the untreated vine shoots, which had the highest content (21.2 ± 0.1 vs. 23.9 ± 0.1 mg g^−1^ DW, respectively).

#### 3.1.2. Stilbene Composition

The stilbene concentration (Rsv and Vf) as affected by each treatment is shown in Table 3. First of all, we determined that the variety influenced the stilbene content. In fact, untreated Negramaro vine shoot extracts contained a higher concentration of Rsv compared to Primitivo (5249.4 vs. 1861.3 mg kg^−1^ DW), while the latter had a higher concentration of Vf (1531.6 vs. 600.1 mg kg^−1^ DW).

It was evident that the drying treatment accounted for some variations in the Rsv and Vf concentrations, according to what we observed for TPC (Table 2). With respect to the Primitivo vine shoots, the drying at 50 °C for 24 h determined the reduction of Rsv (1663.8 ± 16.3 mg kg^−1^ DW) and Vf (1356.8 ± 10.0 mg kg^−1^ DW) when compared to the untreated vine shoots (1861.3 ± 9.8 mg kg^−1^ DW for Rf and 1531.6 ± 89.1 mg kg^−1^ DW for Vf). Minor differences were observed when comparing the other two treatments with the untreated sample: the Rsv concentration increased by only 6.6% after the treatment at 70 °C for 15 min and decreased slightly after the treatment at 80 °C for 10 min (1763.4 ± 98.3 mg kg^−1^ DW); after the treatment at 70 °C for 15 min and 80 °C for 10 min, the concentration of Vf increased by 13% and 5.6%, respectively. Thus, no significant differences were found between the concentrations of Vf after these two treatments. In regards to Negroamaro, significant differences were found among the treatments, with the untreated sample showing the highest Rsv (5249.4 ± 129.8 mg kg^−1^ DW) and Vf concentrations (600.1 ± 79.0 mg kg^−1^ DW) (Table 3).

Overall, these results suggest that the heat pre-treatments either left unchanged or caused a decrease in the stilbene concentration. In particular, the treatments with lower temperatures and longer times led to a significant reduction in Rsv and Vf. Most likely, the use of high temperatures may promote the degradation of some compounds, as reported by Piñeiro et al. [39]. In that case, in most of the selected vine shoots (from 15 grape cane varieties), the total stilbene concentration was significantly higher for freeze-dried extracts than for oven-dried extracts (40 °C for 15 days). However, our results are in contrast with those from Sánchez-Gómez et al. [40], who showed that the thermal treatment led to Rsv concentrations from 6 to 14 times higher than those in the control/no heat treated samples, depending on the vine variety (Airén and Moscatel grape canes).

To the best of our knowledge, this is the first report on the effect of these time-temperature drying parameters on the vine shoot stilbenes contents of Italian vine varieties.

### 3.2. Evaluation of Different Italian Varieties

Considering the results previously obtained, no heat treatment was applied to characterize the stilbene contents in the vine shoots of the investigated Italian varieties. Indeed, in absence of clear advantages, the heat-treatment results are a waste of energy, incompatible with the requests for sustainable processes.

#### 3.2.1. Total Phenolic Content and Antioxidant Activity

The total phenolic contents of the vine shoots are given in Table 4. Vine shoots from the Sangiovese variety showed the lowest TPC, which was approximately 60% lower than Palieri, the variety with the highest content. These results agree with previous studies [50,51]. In fact, Çetin et al. [51], in evaluating the chemical composition of ten different Turkish grape canes varieties, showed that the total phenolic content changed significantly according to the varieties (in a range from 25.36 ± 1.62 to 36.56 ± 2.67 mg GAE g^−1^ DW). Similarly, according to Dorosh et al. [50], the amount of total phenolic content in Tinta Roriz vine shoot extracts (32.6 ± 2.1 mg GAE g^−1^ DW) was 1.6 fold higher than the value obtained from the Touriga Nacional variety (20.1 ± 0.6 mg GAE g^−1^ DW), for the same extraction time and ultrasound extraction technique. These results were in agreement with those from a previous study that also presented a summary table showing the results from selected published papers examining the phenolic compounds of vine shoots extractions [48].

Table 4 shows the antioxidant properties of the extracts from the vine shoots of the Italian varieties evaluated. The antioxidant activity showed statistically significant differences among the varieties with the same tendency as that previously described for TPC. As reported in Table 5 and as previously demonstrated, antioxidant activity correlates with the total phenolic content of grape cane extracts [52]. These results were quite consistent with those provided by the DPPH assay, since the Palieri, Montepulciano, and Notardomenico vine shoot extracts showed the highest antioxidant capacity (112.1 ± 0.6, 111.6 ± 0.8, and 115.2 ± 1.2 µmol TE g^−1^ DW, respectively) and, at the same time, the highest total phenolic content. Additionally, according to the ABTS assay, the Montepulciano vine shoot extracts had the highest antioxidant activity (156.4 ± 0.8 µmol TE g^−1^ DW). It is very difficult to compare the results obtained from this characterisation with those from other studies because most of those used different assays to evaluate the antioxidant activity. Nevertheless, some researchers have compared the antioxidant activity of vine shoot extracts of different varieties [45,49,50]. For example, Guerrero et al. [18] found significant differences in the antioxidant activity of the vine shoots from 22 grape varieties (including *Vitis vinifera sativa*, *Vitis vinifera sylvestris,* and hybrid direct producers), measured using the oxygen radical absorbance capacity (ORAC) assay (range from 1700 to 5300 µmol, Trolox equivalent g^−1^ DW).

#### 3.2.2. Stilbene Composition

**Figure 1:**
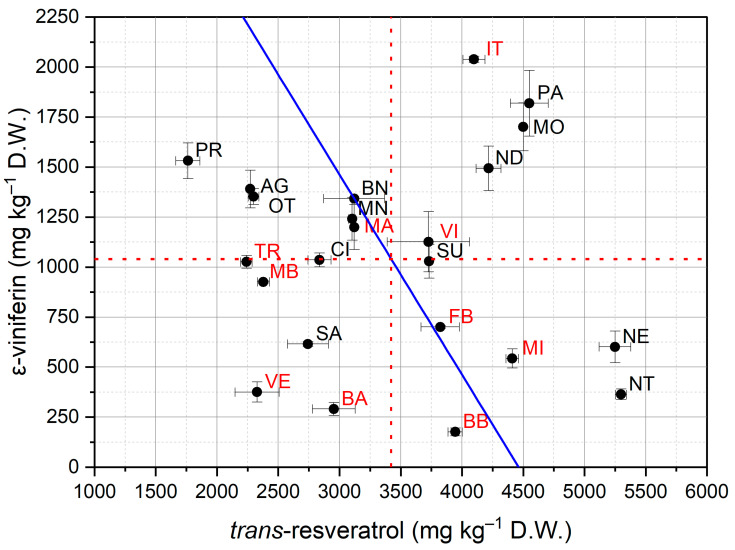
The stilbene contents in vine shoots from 23 different Italian varieties. Red dotted lines—the mean contents of *trans*-resveratrol (Rsv) and ε-viniferin (Vf); blue solid line—the mean content of *trans*-resveratrol + ε-viniferin. Black labels indicate black grape varieties; red labels indicate white grape varieties. For sample codes, see Table 1.

Figure 1 shows the stilbene concentration of the vine shoot extracts of the investigated varieties while the Rsv, Vf, and total stilbenes concentrations (mean ± standard deviation) are reported in Table S1 (Supplementary Material). The mean total concentration of stilbenes, approximately 4500 mg kg^−1^ DW, varied greatly depending on the variety, with values ranging between 2700 and 6400 mg kg^−1^ DW for Verdeca and Palieri, respectively, with 2.4-fold higher results for the latter. Nevertheless, the Palieri, Montepulciano, and Italia varieties presented the highest total stilbene concentration. In contrast, the Verdeca, Bianco d’Alessano, and Trebbiano varieties presented the lowest total stilbene concentration. In previous studies, a wide variability (from 2.5 to 4-fold) of total polyphenol amounts was already observed among different vine shoot varieties [18,30,37].

The major stilbene compounds found in all the collected samples were Rsv (mean of 3422.2 mg kg^−1^ DW), followed by Vf (mean of 1040.0 mg kg^−1^ DW). An example of HPLC-DAD chromatograms of the Palieri vine shoot extract is provided in the Supplementary Material Figure S1. These results agree with those observed in the studies by Vergara et al. [29] and Gorena et al. [33], in which the major stilbene compound found in most grape cane extracts considered were Rsv. In contrast, according to Guerrero et al. [18,32] and Lambert et al. [30], Vf was the most abundant compound in vine shoots of different *Vitis* varieties.

The highest mean concentration of Rsv was determined for the Nero di Troia (5298.1 ± 45.2 mg kg^−1^ DW) and Negroamaro vine shoots (5249.4 ± 129.8 mg kg^−1^ DW), followed by the Montepulciano and Palieri varieties. On the other hand, the Primitivo vine shoots (1861.3 ± 9.8 mg kg^−1^ DW) showed the lowest concentration of Rsv, about 64.9% less than Nero di Troia. There are many studies showing the differences between the stilbene contents in vine shoots from different varieties and species of vines [26,32], but there are no studies concerning the variation in vine shoots of these Italian varieties. Nevertheless, comparable concentrations of Rsv were found in vine shoots of different Chilean varieties, in which Gewurztraminer (mean 4628 ± 568 mg kg^−1^ DW) and Pinot noir varieties (mean 3676 ± 353 mg kg^−1^ DW) were determined to contain high levels of this compound [29]. Recently, Zwingelstein et al. [31] showed that vine shoots of the Mondeuse variety contained higher levels of Rsv (3759–4636 mg kg^−1^ DW) than those of the Jacquère variety (2259–2994 mg kg^−1^ DW). Lower concentrations were found by Zhang et al. [28], in which the *Vitis Vinifera* vine shoots grown in China exhibited an Rsv content ranging from 664 to 1751 mg kg^−1^.

In regard to Vf, Figure 1 clearly reveals that most vine shoot extracts of red berry varieties had a concentration of Vf above the average. Nevertheless, the highest concentration was found in the vine shoot extracts of the Italia variety (2038.4 ± 15.8 mg kg^−1^ DW), when compared to other varieties. The Bombino Bianco variety (175.9 ± 19.6 mg kg^−1^ DW) showed a concentration 91.37% lower than that of the Italia variety. These results agreed with those observed in the studies by Guerrero et al. [18] in which the highest concentration of Vf, found in Gewurztraminer (2810.4 mg kg^−1^ DW), was similar to that found in this study. Similarly, according to Lambert al. [30], the most abundant stilbenoid in grape canes of sixteen *Vitis Vinifera* varieties was Vf (mean of 2171 mg kg^−1^ DW).

To evaluate the correlation between the TPC and the concentration of Rsv and Vf, the Pearson’s correlation coefficient test was applied (Table 6). A significant correlation between TPC and Rsv and between TPC and Vf was observed, whilst no correlation was found between the two considered stilbenes (*p* = 0.697). A clear explanation for this absence of correlation is difficult to determine, considering that several sources of variability could affect the stilbene synthesis and outcome. From the genetic point of view, stilbene synthase (STS) is the key enzyme in the stilbene biosynthetic pathway, and grapevines contain a large number of STS genes [53,54]. Moreover, as reported in a recent review [11], the expression of these genes also varies according to environmental stress. At the same time, the specific varieties affect the accumulation of stilbenes, even under the same environmental conditions [32,34]. Vf is an oligomer of Rsv that accumulates in plants by oxidative coupling, affected by different biotic and abiotic stresses [11,55]. Thus, it could be supposed that Vf accumulation is independent of the original Rsv content, yet much more correlated to environmental the stresses on the plant material.

## 4. Conclusions

Vine shoots are a rich source of bioactive compounds, with Rsv and Vf stilbenes characterised as the most important. The amounts of these stilbenes in the vine shoots could be strongly affected by both extrinsic and intrinsic factors. Our results showed that the heat pre-treatment of the plant material had a negligible effect on the concentration of TPC, Rsv, and Vf. On the other hand, the genotype had a strong influence on Rsv and Vf accumulation. The results of this work confirmed the possibility of obtaining extracts particularly rich in Rsv from Italian vine shoots, assigning an important economic value to a waste product with zero cost.

Thanks to its many applications, resveratrol has great potential in the future market. A recent report shows that the global resveratrol market will reach USD 99.4 million by the end of 2026 [56]. However, its price also depends on the costs of the raw materials and the entire extraction process. Considering this last point, the outcomes of this work impart useful insights proving that there is no need to consume energy for vine shoot pre-treatment, decreasing the general costs. However, more studies are needed to confirm these observations and to investigate the concentration of Rsv and other stilbene compounds in the same vine shoot varieties from different geographical areas or in other Italian varieties.

## Figures and Tables

**Table 1:** The grapevine variety name, grape colour, usual use, and acronym used in the text.

Variety	Colour	Use	Acronym
Aglianico	red	wine	AG
Bianco d’Alessano	white	wine	BA
Bombino Bianco	white	wine	BB
Bombino Nero	red	wine	BN
Ciliegiolo	red	wine	CI
Fiano Bianco d’Avellino	white	wine	FB
Italia	white	table	IT
Malvasia Bianca	white	wine	MB
Malvasia Nera di Brindisi	red	wine	MN
Maresco Bianco	white	wine	MA
Minutolo Bianco	white	wine	MI
Montepulciano	red	wine	MO
Negroamaro	red	wine	NE
Nero di Troia	red	wine	NT
Notardomenico	red	wine	ND
Ottavianello	red	wine	OT
Palieri	red	table	PA
Primitivo	red	wine	PR
Sangiovese	red	wine	SA
Susumaniello	red	wine	SU
Trebbiano	white	wine	TR
Verdeca	white	wine	VE
Vittoria	white	table	VI

**Table 2:** The TPC (total phenolic content), DPPH (antioxidant activity determined by the DPPH assay), and ABTS (antioxidant activity determined by the ABTS assay) of the vine shoot extracts from the Primitivo and Negroamaro varieties. Results are expressed as mean ± standard deviation (*n* = 2); different letters for each variety in the same column indicate a significant difference according to the Fisher test (*p* < 0.05).

Sample	TPC (mg GAE g^−1^ DW)	DPPH (µmol TE g^−1^ DW)	ABTS (µmol TE g^−1^ DW)
Primitivo			
Untreated	20.1 ± 0.1 ^a^	85.0 ± 4.1 ^a^	115.6 ± 1.6 ^a^
50 °C–24 h	18.4 ± 0.1 ^b^	80.6 ± 1.3 ^ab^	97.0 ± 0.7 ^d^
70 °C–15 min	21.1 ± 0.6 ^a^	78.2 ± 0.1 ^b^	101.2 ± 0.8 ^c^
80 °C–10 min	20.3 ± 0.6 ^a^	81.8 ± 0.9 ^ab^	110.8 ± 0.8 ^b^
Negroamaro			
Untreated	23.9 ± 1.0 ^a^	79.5 ± 0.2 ^a^	136.5 ± 0.8 ^a^
50 °C–24 h	21.8 ± 0.1 ^bc^	57.9 ± 0.5 ^d^	86.4 ± 0.9 ^c^
70 °C–15 min	21.2 ± 0.1 ^c^	63.2 ± 0.4 ^c^	79.2 ± 0.7 ^d^
80 °C–10 min	22.8 ± 0.1 ^ab^	65.0 ± 0.7 ^b^	88.7 ± 0.7 ^b^

**Table 3:** The stilbene concentrations in Primitivo and Negramaro vine shoot extracts. The means and standard deviation (*n* = 2) are represented in the same column and different letters for each variety indicate significant differences (*p* < 0.05).

	Stilbene Concentrations (mg kg^−1^ DW)	
Sample	*Trans*-Resveratrol	ε-Viniferin
Primitivo		
Control	1861.3 ± 9.8 ^ab^	1531.6 ± 89.1 ^ab^
50 °C–24 h	1663.8 ± 16.3 ^c^	1356.8 ± 10.0 ^c^
70 °C–15 min	1983.8 ± 12.9 ^a^	1731.2 ± 56.0 ^a^
80 °C–10 min	1763.4 ± 98.3 ^bc^	1617.5 ± 13.1 ^a^
Negroamaro		
Control	5249.4 ± 129.8 ^a^	600.1 ± 79.0 ^a^
50 °C–24 h	4471.1 ± 73.9 ^b^	451.5 ± 1.0 ^c^
70 °C–15 min	4626.0 ± 37.7 ^b^	455.7 ± 9.8 ^c^
80 °C–10 min	4925.4 ± 14.4 ^ab^	525.2 ± 38.2 ^b^

**Table 4:** The total phenolic content and antioxidant activity of vine shoot extracts from 23 different Italian varieties. Means and standard deviation (*n* = 2) are represented in the same column and data followed by different letters indicate statistically significant differences according to the Fisher test (*p* < 0.05). For sample codes, see Table 1.

Sample	TPC (mg GAE g^−1^ DW)	DPPH (µmol TE g^−1^ DW)	ABTS (µmol TE g^−1^ DW)
AG	18.0 ± 0.3 ^jk^	72.2 ± 2.1 ^hijk^	102.2 ± 2.8 ^hi^
BA	24.4 ± 0.2 ^de^	90.6 ± 7.2 ^c^	137.2 ± 11.7 ^bc^
BB	17.8 ± 0.2 ^jk^	69.4 ± 2.4 ^ijk^	116.2 ± 1.2 ^efg^
BN	22.5 ± 1.8 ^efg^	92.9 ± 1.8 ^bc^	135.0 ± 3.2 ^bc^
CI	19.6 ± 0.5 ^hij^	37.4 ± 0.8 ^l^	34.2 ± 0.7 ^k^
FB	20.4 ± 0.1 ^ghi^	86.3 ± 0.0 ^cde^	79.7±1.4 ^j^
IT	27.6 ± 1.1 ^bc^	84.9 ± 2.9 ^cdef^	144.1 ± 16.4 ^b^
MB	19.6 ± 1.3 ^hij^	75.6 ± 2.1 ^ghij^	75.7 ± 1.4 ^j^
MN	21.3 ± 1.2 ^fgh^	76.0 ± 8.0 ^ghij^	121.8 ± 7.5 ^def^
MA	23.0 ± 1.5 ^def^	87.9 ± 1.9 ^cd^	125.9 ± 0.2 ^cde^
MI	22.4 ± 0.7 ^efg^	77.4 ± 3.9 ^fghi^	132.6 ± 4.2 ^cd^
MO	28.6 ± 0.8 ^b^	111.6 ± 0.8 ^a^	156.4 ± 0.8 ^a^
NE	23.9 ± 1.0 ^de^	79.5 ± 0.2 ^efgh^	136.5 ± 0.8 ^bc^
NT	24.2 ± 2.0 ^de^	81.2 ± 3.9 ^defg^	103.0 ± 4.3 ^hi^
ND	29.3 ±1.7 ^b^	115.2 ± 1.2 ^a^	112.6 ± 2.7 ^fgh^
OT	18.8 ± 1.0 ^ijk^	70.4 ± 2.3 ^ijk^	108.4 ± 2.0 ^ghi^
PA	36.9 ± 2.2 ^a^	112.1 ± 0.6 ^a^	131.5 ± 3.9 ^cd^
PR	20.1 ± 0.1 ^ghij^	85.0 ± 4.1 ^cdef^	115.6 ± 1.6 ^efg^
SA	14.7 ± 0.2 ^l^	67.2 ± 1.4 ^k^	108.4 ± 1.3 ^ghi^
SU	22.4 ± 1.3 ^efg^	91.7 ± 7.7 ^c^	99.7 ± 3.8 ^i^
TR	18.9 ± 0.1 ^hijk^	72.0 ± 7.6 ^hijk^	108.0 ± 3.5 ^ghi^
VE	17.4 ± 1.4 ^k^	69.2 ± 5.9 ^jk^	108.4 ± 7.2 ^ghi^
VI	25.1 ± 1.3 ^cd^	100.5 ± 1.9 ^b^	111.8 ± 8.6 ^fgh^

**Table 5:** The Pearson’s correlation coefficients between the TPC, ABTS, and DPPH in 23 vine shoot extracts.

	TPC	ABTS	DPPH
TPC	1	-	-
ABTS	0.450 (*p* = 0.002)	1	-
DPPH	0.760 (*p* < 0.001)	0.606 (*p* < 0.001)	1

**Table 6:** The Pearson’s correlation coefficients between the TPC, *trans*-resveratrol, and ε-viniferin in 23 vine shoot extracts.

	TPC	*Trans*-Resveratrol	ε-Viniferin
TPC	1	-	-
*trans*-resveratrol	0.626 (*p* < 0.001)	1	-
ε-viniferin	0.515 (*p* < 0.001)	−0.059 (*p* = 0.697)	1

## Data Availability

Not applicable.

## Supplementary Materials

The following supporting information can be downloaded at: https://www.mdpi.com/article/10.3390/foods11040553/s1, Figure S1: Stilbenes HPLC-DAD chromatogram of cultivar Palieri vine-shoots extract detected at 306 nm (a) and 324 nm (b); Table S1: Stilbene concentrations (mg kg^−1^ DW) in vine-shoots from 23 different Italian varieties. Means and standard deviation (*n* = 2) are represented in the same column and data followed by different letters indicate statistically significant differences according to Fisher’s LSD test (*p* < 0.05). For sample codes see Table 1 of the main text.
