# Supplementary material for: Vine Shoots as a Source of Trans-Resveratrol and ε-Viniferin: A Study of 23 Italian Varieties
Source: Foods. 2022 Feb 15;11(4):553. doi: 10.3390/foods11040553 (PMC8871016; doi:10.3390/foods11040553)
Supplement: Supplementary file 1 [file foods-11-00553-s001.zip › foods-1535546-supplementary.pdf]

Article

Vine-shoots as a source of *trans*-resveratrol and ε-viniferin: a study about 23 Italian cultivar

Mirella Noviello, Antonio Francesco Caputi, Giacomo Squeo, Vito Michele Paradiso, Giuseppe Gambacorta and Francesco Caponio*

*Correspondence: Department of Soil, Plant and Food Science (DISSPA), University of Bari Aldo Moro, via Amendola, 165/a, I-70126 Bari, Italy; Francesco Caponio francesco.caponio@uniba.it;

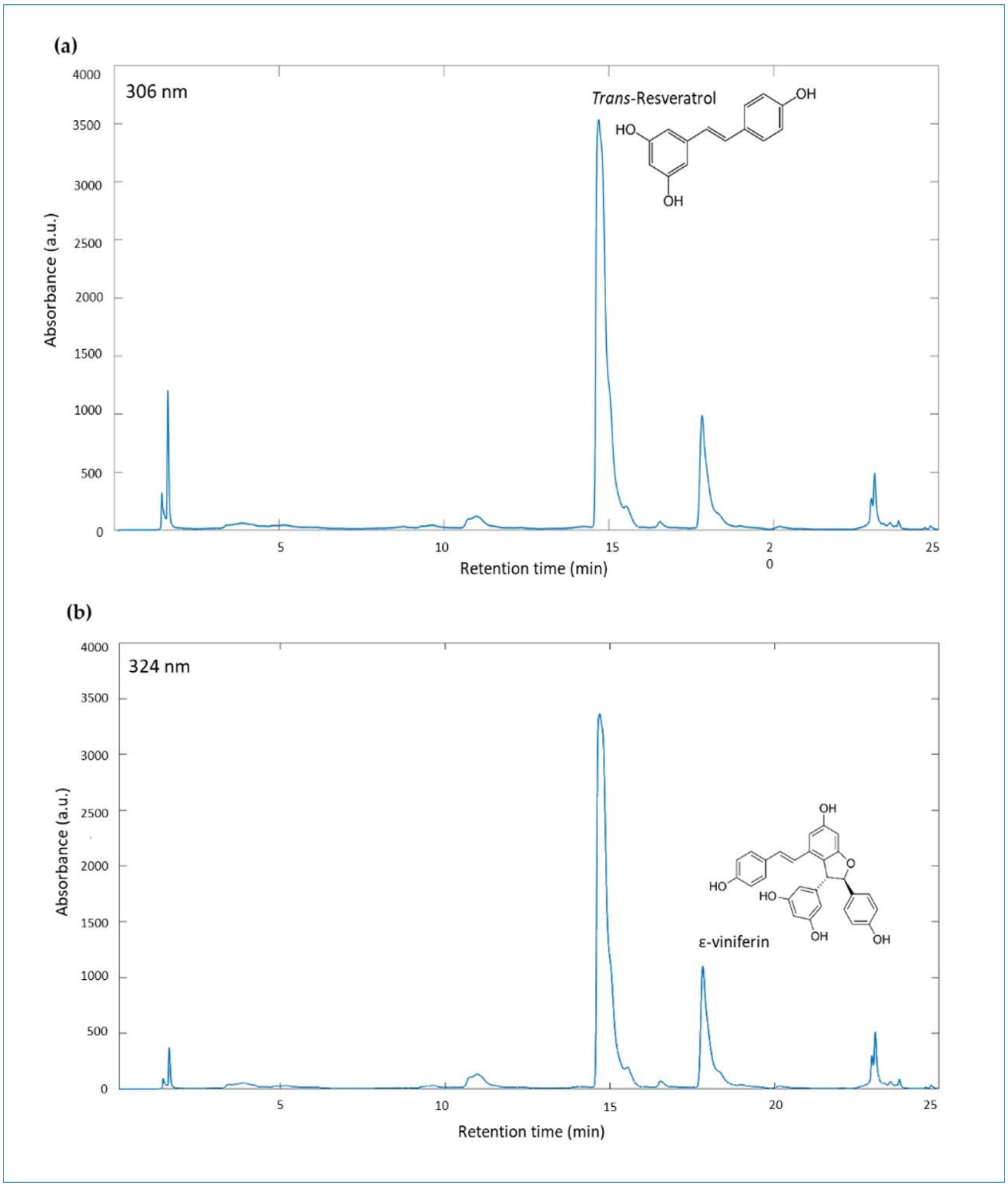

Figure S1. Stilbenes HPLC-DAD chromatogram of cultivar Palieri vine-shoots extract detected at 306 nm (a) and 324 nm (b).

Table S1. Stilbene concentrations (mg kg^{-1} DW) in vine-shoots from 23 different Italian varieties. Means and standard deviation (n=2) are represented in the same column and data followed by different letters indicate statistically significant differences according to Fisher's LSD test ($P < 0.05$). For sample codes see table 1 of the main text.

	**Stilbene concentrations (mg kg^{-1} DW)**		
**Sample**	***trans*-resveratrol**	**ε-viniferin**	**Total stilbenes**
AG	2299.1 ± 44.6^{j}	1352.4 ± 39.6^{def}	3651.5 ± 5.0^{jkl}
BA	2954.0 ± 173.8^{ghi}	290.8 ± 32.0^{kl}	3244.8 ± 205.8^{m}
BB	3945.0 ± 57.6^{ef}	175.9 ± 19.6^{l}	4121.0 ± 38.0^{hi}
BN	3120.4 ± 248.6^{g}	1342.7 ± 0.5^{def}	4463.1 ± 248.1^{fgh}
CI	2837.3 ± 94.4^{hi}	1035.9 ± 34.5^{hi}	3873.3 ± 128.9^{ij}
FB	3823.2 ± 156.3^{f}	700.4 ± 7.0^{j}	4523.6 ± 149.3^{efg}
IT	4098.0 ± 90.5^{de}	2038.4 ± 15.8^{a}	6136.5 ± 74.6^{ab}
MB	2378.9 ± 48.7^{j}	926.1 ± 6.0^{i}	3305.1 ± 42.6^{klm}
MN	3103.7 ± 27.6^{ghi}	1240.9 ± 106.8^{efg}	4344.6 ± 134.5^{gh}
MA	3120.7 ± 12.9^{g}	1199.8 ± 112.9^{fg}	4320.6 ± 101.6^{gh}
MI	4410.9 ± 51.4^{bc}	543.1 ± 48.0^{j}	4954.0 ± 99.3^{d}
MO	4500.6 ± 0.1^{b}	1701.1 ± 119.7^{b}	6201.7 ± 119.9^{ab}
NE	5249.4 ± 129.8^{a}	600.9 ± 79.0^{j}	5850.4 ± 50.8^{bc}
NT	5298.1 ± 45.2^{a}	363.5 ± 26.8^{k}	5661.6 ± 18.4^{c}
ND	4217.2 ± 101.5^{cd}	1493.6 ± 111.2^{cd}	5710.8 ± 212.8^{c}
OT	2271.8 ± 17.6^{j}	1390.4 ± 94.2^{cde}	3662.2 ± 111.9^{jk}
PA	4549.6 ± 153.8^{b}	1819.3 ± 164.3^{b}	6369.0 ± 318.1^{a}
PR	1861.3 ± 9.8^{k}	1531.6 ± 89.1^{c}	3392.9 ± 98.9^{lm}
SA	2742.6 ± 166.5^{i}	615.6 ± 12.4^{j}	3358.2 ± 154.0^{klm}
SU	3731.8 ± 11.6^{f}	1029.2 ± 85.0^{hi}	4761.0 ± 73.4^{def}
TR	2240.2 ± 47.5^{j}	1029.3 ± 32.6^{hi}	3266.6 ± 80.0^{m}
VE	2327.4 ± 179.7^{j}	375.4 ± 50.7^{k}	2702.9 ± 230.4^{n}
VI	3726.2 ± 336.0^{f}	1126.0 ± 150.5^{gh}	4852.2 ± 486.6^{de}
